# Use of the dual tyrosine kinase inhibitor lapatinib for the treatment of vulvovaginal squamous cell carcinoma in a dog

**DOI:** 10.1093/jvimsj/aalag116

**Published:** 2026-06-18

**Authors:** Mark J Vardanega, Tiffany Longo, Alex Aceino, Caleb K Alexander

**Affiliations:** Ocean State Veterinary Specialists, East Greenwich, RI, United States; Ocean State Veterinary Specialists, East Greenwich, RI, United States; Antech Diagnostics, Middletown, CT, United States; Ocean State Veterinary Specialists, East Greenwich, RI, United States

**Keywords:** dual tyrosine kinase inhibitor, epidermal growth factor receptor inhibitor, lapatinib, vulvovaginal neoplasia

## Abstract

Vulvovaginal squamous cell carcinoma (SCC) is an uncommon neoplasm in dogs. An 11-year-old female spayed German Shepherd dog presented with a history of excessive peri-vulvar grooming and a 3 cm × 3 cm irregular mass involving the vagina and vulva. Incisional biopsy confirmed a diagnosis of primary vulvovaginal SCC. Abdominal ultrasonography and thoracic radiographs indicated no evidence of systemic or regional metastasis. Surgical excision and adjuvant radiation therapy were declined, and the patient was treated with lapatinib, an orally administered dual tyrosine kinase inhibitor. Treatment resulted in a partial response within 1 month and complete resolution of the primary lesion within 3 months. No recurrence of the initial lesion was observed after 1 year of treatment despite development of perivulvar cutaneous lesions that were not associated with clinical signs. Lapatinib was well tolerated but did result in acute hepatic injury that was resolved using hepatoprotective antioxidant medication and intermittent treatment cessation.

## Introduction

Squamous cell carcinoma (SCC) is a malignant neoplasm that arises from keratinocytes in dogs, commonly occurring in the skin, oral cavity, nasal cavity, lung, and bladder.^1^ Primary vulvovaginal SCC is common in humans but uncommonly reported in dogs, and has only been clinically described in 2 cases.^2–4^

Vulvovaginal SCC in dogs is locally invasive with the potential to spread to the urinary tract, inguinal skin of the ventrum, and regional lymph nodes.^4^ Wide surgical excision with vulvo-vaginectomy and prepubic urethrostomy is the treatment of choice in dogs with malignant vulvovaginal neoplasia.^4^^,^^5^ Prognosis after wide surgical excision of vulvovaginal SCC is reportedly poor in dogs, with a survival time of 4-8 months and disease free interval of 1 month.^4^

In our case, surgery and radiation therapy were declined and medical treatment using an orally administered small molecule inhibitor (lapatinib) was pursued. Lapatinib is a dual inhibitor of epidermal growth factor receptor (EGFR) and human epidermal growth factor receptor 2 (HER2) tyrosine kinases. The treatment of primary vulvovaginal squamous cell carcinoma using lapatinib has not been previously reported in dogs.

## Case history

**Figure 1:**
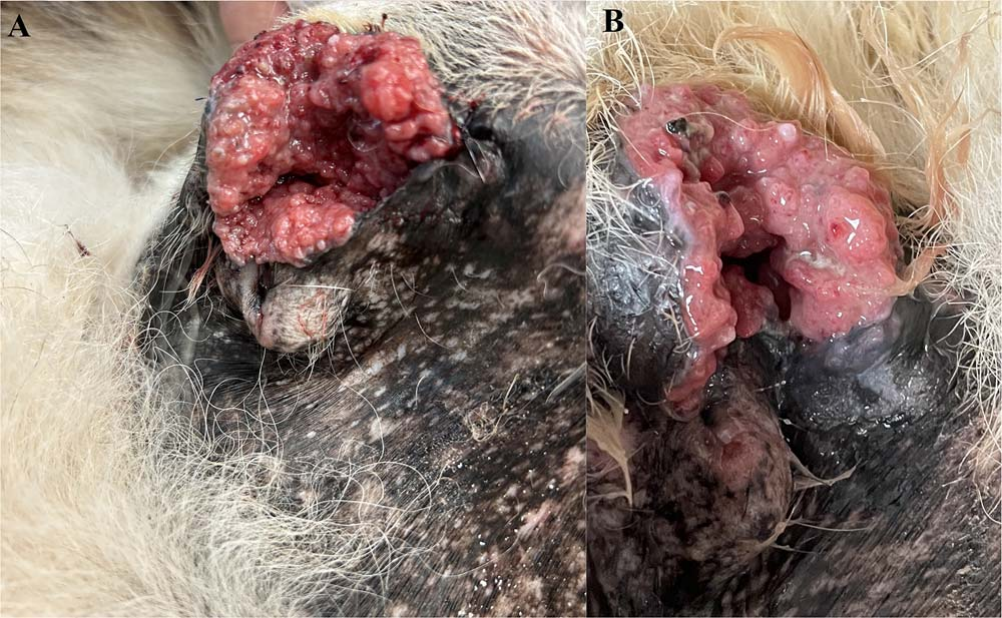
Vulvovaginal squamous cell carcinoma lesion at the time of incisional biopsy. (A) Photograph of the 3 cm diameter, irregular vulvovaginal mass extending across the right side of the vulva. (B) Photograph of the 3 cm diameter, irregular vulvovaginal mass arising from the vagina and extending across the right side of the vulva.

An 11-year-old, 44.5 kg, female spayed German Shepherd dog initially presented to the primary care veterinarian with a history of licking at the vulva and a mass around the perineum. Physical examination disclosed a raised, firm mass with a moist ulcerated defect and central pocket extending from the right cranial aspect of the vulva and vagina (Figure 1). Cytologic evaluation of an impression smear featured neutrophilic inflammation and abundant cocci and rod bacteria. The lesion was cleansed using dilute chlorhexidine solution, and the dog was treated with amoxicillin-clavulanic acid (14 mg/kg PO q12h for 7 days).

**Figure 2:**
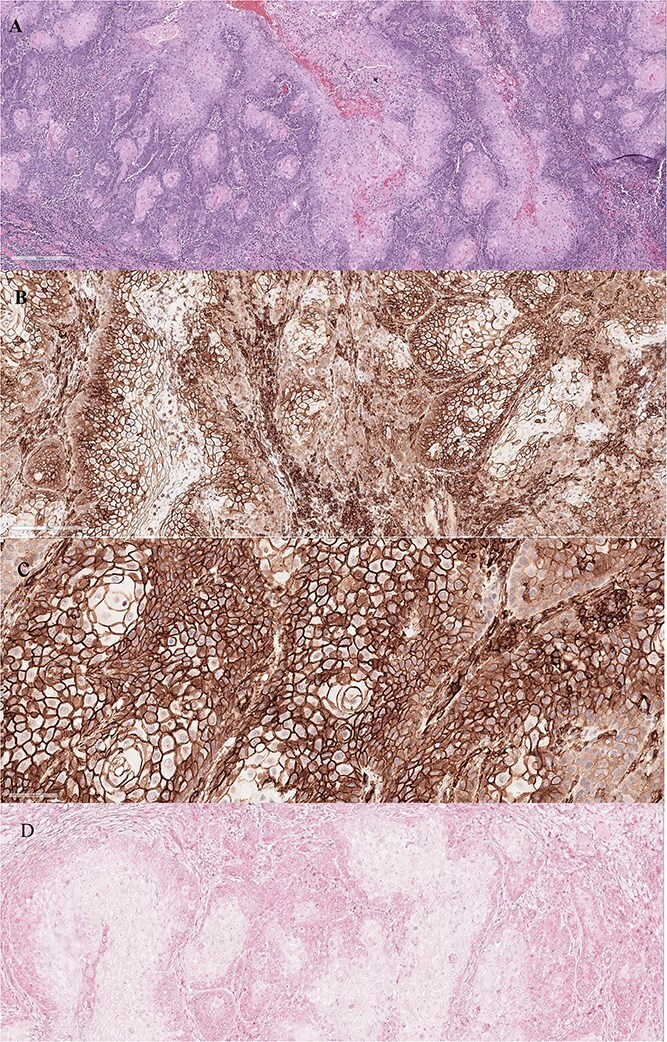
**(**A) Hematoxylin and eosin-stained sections of the paraffin embedded biopsy. Histopathology indicated a dense population of neoplastic squamous epithelial cells infiltrating the dermis and submucosa. Neoplastic cells display increased atypia and mitotic activity along with evidence of dyskeratosis. A large amount of neutrophilic to lymphoplasmacytic inflammation is associated with the neoplastic cells. Scale bar = 300 μm. 96× magnification. (B) Immunohistochemistry staining of biopsy demonstrating neoplastic cells show membranous positivity for EGFR. Scale bar = 200 μm. 19× magnification. (C) Immunohistochemistry staining of biopsy material demonstrating neoplastic cells with membranous positivity for EGFR. Scale bar = 60 μm. 40× magnification. (D) Immunohistochemistry staining of biopsy material, neoplastic cells display rare, and faint membranous labeling with HER2 (<10% of neoplastic cells) and minimal cytoplasmic labeling interpreted as a negative result. Scale bar = 200 μm. 20× magnification.

Two incisional biopsy samples were collected 7 days later under sedation and using a 6 mm skin biopsy punch. Preoperative hematology and serum biochemistry results were unremarkable. Histopathologic assessment indicated a poorly demarcated, densely cellular, unencapsulated invasive neoplasm consisting of keratinocytes forming nests, islands and cords, and supported by a dense fibrous stroma, consistent with squamous cell carcinoma (Figure 2). A mitotic count of 69 per 10 high powered fields was reported without angiolymphatic invasion. Neoplastic cells featured dyskeratosis with large numbers of inflammatory cells present, and the surface of the mass was ulcerated and covered with necrotic debris, hemorrhage, and fibrin.

On immunohistochemistry (IHC), the neoplastic cells had diffusely positive membranous labeling for EGFR (Figure 2). Rare and faint membranous labeling for HER2 (<10% of neoplastic cells) was interpreted as a negative result (Figure 2).

The dog was referred to an oncologist 2 weeks after initial presentation. Physical examination confirmed the presence of a 3 cm diameter, raised, proliferative, ulcerated mass in the perivulvar region (Figure 1). Abdominal ultrasonography and thoracic radiography were performed and showed no evidence of distant or regional metastasis. Surgical resection and adjuvant radiation therapy were recommended. Alternative options including radiation therapy alone, cytotoxic chemotherapy, or lapatinib were discussed. The owners declined surgical resection and radiation therapy and elected treatment with lapatinib (1000 mg [22.5 mg/kg] PO q24h).^6^ Lapatinib was proposed because of its action as a dual inhibitor of EGFR and HER2, both of which are commonly present in numerous solid tumors including cutaneous SCC in dogs, its documented in vitro and in vivo activity against urothelial carcinoma, and the positive EGFR finding on IHC.^6^^,^^7^

The dog was re-evaluated 4 weeks after starting lapatinib and a marked decrease in tumor size was observed. The large, ulcerated mass extending from the vulva was almost not visible, but 2 new 2 mm diameter circular pink masses were observed in the right perivulvar region. Repeat hematology was normal, but serum biochemistry disclosed markedly increased alanine aminotransferase (ALT) activity (1046 U/L). Because of the risk of hepatotoxicity, lapatanib was discontinued and Denamarin (635 mg s-adenosyl-L-methionime and 180 mg silybin PO q24h) and α lipoic acid (1000 mg PO q12h) were started. Repeat laboratory testing 2 weeks later indicated normalization of the serum ALT activity. Lapatinib was restarted after having been discontinued for a total of 16 days and hepatoprotective treatment was continued.

**Figure 3:**
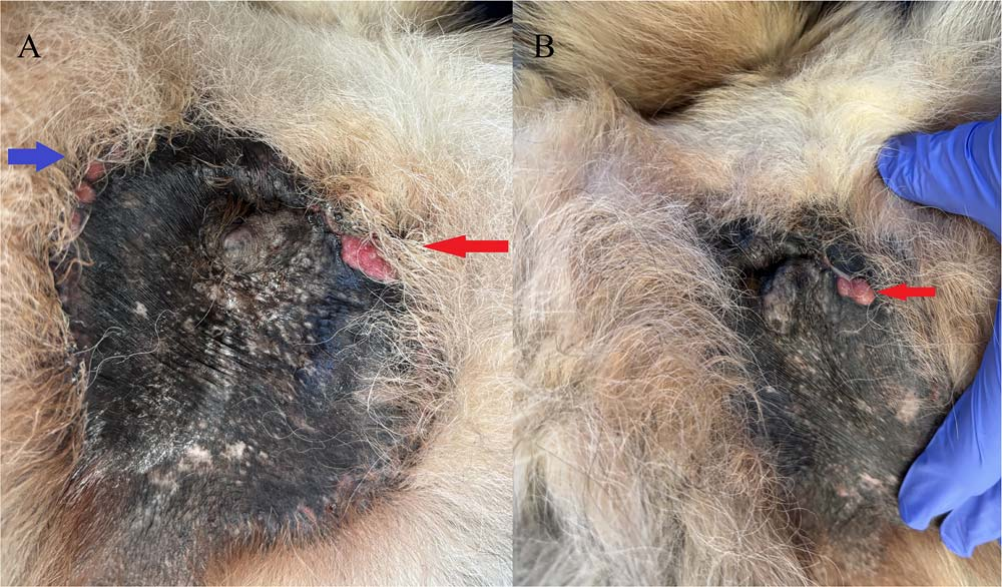
(A) Photograph of the vulva taken thirteen weeks after starting lapatinib treatment. The primary lesion has completely regressed. Blue arrow points to 3 small 3 mm diameter raised pink cutaneous lesions on the left side of the vulva. Red arrow points to a single 10 × 5 mm raised elliptical cutaneous lesion on the right side of the vulva. Cutaneous peri-vulvar lesions represent suspected local cutaneous metastasis. (B) Photograph of the vulva taken 21 weeks after starting lapatinib treatment. Primary lesion and left sided peri-vulvar lesions have completely regressed. Red arrow points to 10 × 5 mm raised cutaneous lesion on the right of the vulva.

At re-assessment 13 weeks after initially starting lapatinib, the vulvar mass could not be identified. A single 1 × 0.5 cm pink, slightly raised elliptical lesion in the peri-vulvar skin on the right and 3 small 3 mm pink raised, cutaneous lesions on the left side of the vulva were noted (Figure 3). Repeat hematology and serum biochemistry results were normal. Cytology of a fine needle aspirate of the lesion on the right lateral aspect of vulva identified numerous neutrophils, cocci, and rafts of epithelial cells with mild anisocytosis suggestive of carcinoma and local cutaneous metastasis. The dog was started on cefpodoxime (300 mg PO q24h for 7 days) and topical Neosporin, and α lipoic acid was discontinued.

Reevaluation was performed 21 weeks after initially starting lapatinib. The previously noted ulcerated mass protruding from the vulva and cutaneous lesions to the left of the vulva were not visible and a 6 mm × 8 mm diameter pink, oblong lesion was observed to the right of the vulva (Figure 3). Subsequent reevaluations performed at 32 and 42 weeks after commencement of treatment showed no substantial change in the size and number of the perivulvar lesions. Repeat hematology and serum biochemistry identified no abnormalities at all time points and Denamarin was discontinued at the 32-week reevaluation. Thoracic radiographs were performed 42 weeks after the start of treatment that showed no evidence of pulmonary metastatic disease. At the time of publication lapatinib treatment was ongoing and no local recurrence of the primary vulvovaginal mass has been detected and no clinical signs associated with the perivulvar lesions have been noted.

## Discussion

Primary vaginal and vulvar tumors are relatively uncommon in dogs, representing 1.6%-3% of all canine neoplasms, and they are benign in 73%-94% of cases.^2^^,^^3^^,^^5^ Primary vulvovaginal SCC is uncommon in dogs, and clinical management and outcome have only been reported in 2 cases.^4^

Treatment for SCC typically relies on surgical excision to obtain local control. However, alternative or adjunctive treatment using photodynamic therapy, non-steroidal anti-inflammatory drugs (NSAIDs), retinoids, chemotherapy, and radiation therapy also has been described.^1^^,^^4^^,^^8–14^

Curative intent surgical excision of malignant vulvar and vaginal tumors can include vulvo-vestibulectomy, vulvo-vestibulo-vaginectomy, partial vaginectomy, complete vaginectomy, partial vestibulo-vaginectomy, and urethroplasty.^5^^,^^15^ Surgery is reportedly well-tolerated in dogs but is associated with a high complication rate of up to 38%, including a 28% incidence of postoperative urinary incontinence.^5^

Curative intent surgical excision of vulvovaginal SCC using a combined vulvo-vaginectomy and prepubic urethrostomy has been described.^4^ Clean surgical margins were obtained in both cases but local recurrence occurred within 1 month of surgery despite use of adjuvant chemotherapy with NSAIDs (*n* = 2), cyclophosphamide (*n* = 1), and toceranib (*n* = 1).^4^

In our case, the owners declined surgical excision because of lesion size, patient age, high complication rate, and the invasive nature of surgery. Instead, the owners elected medical treatment using lapatinib.

Lapatinib is a dual inhibitor of EGFR and HER2 tyrosine kinases.^16^^,^^17^ These receptors are commonly over-expressed in several solid tumors, including cutaneous canine squamous cell carcinoma. Immunohistochemistry confirmed positive expression of EGFR in our case (Figure 2).^6^^,^^7^ Lapatinib blocks the adenosine triphosphate binding site of the intracellular domains of HER2 and EGFR resulting in tumor growth inhibition, and is typically well tolerated with minor adverse effects.^16^^,^^17^ In vitro studies have indicated anti-tumor effects of lapatinib against prostatic carcinoma, transitional cell carcinoma (urothelial carcinoma), and mammary carcinoma in dogs.^18–20^

A recent non-randomized clinical trial in 86 dogs with muscle-invasive urothelial carcinoma indicated that treatment with lapatinib and piroxicam was superior to piroxicam alone.^6^ Adverse effects were reported in 82% of cases but all were mild to moderate in severity, with lapatinib-treated dogs showing a higher incidence of increased ALT and ALP activity.^6^

The use of lapatinib for SCC in dogs has not been reported, but it has been evaluated in cutaneous and vulvar SCC in humans and demonstrated a dramatic clinical effect in some cases.^21^^,^^22^ Evaluation in larger clinical trials has shown that lapatinib is well tolerated but has inconsistent efficacy in cutaneous SCC in humans.^23^ A similar EGFR inhibitor, erlotinib, has been evaluated in humans with vulvar SCC and had a clinical effect in 67.5% of cases with 27.5% experiencing partial response and 40% experiencing stable disease.^24^ Lapatinib treatment in our patient resulted in a marked partial response within 1 month and complete resolution of the initial vulvar lesion within 3 months.

The metastatic rate for SCC in dogs varies based on anatomic location ranging from 5% to 22% for dermal SCC and up to 60% for tonsillar SCC.^1^^,^^11^^,^^13^^,^^14^^,^^25^ Vulvovaginal SCC appears to be more aggressive with metastasis occurring in reported cases in dogs, including local invasion into the urinary tract (*n* = 2) and ventro-inguinal skin (*n* = 1); and regional lymph node metastasis (*n* = 2).^4^ In our case, no evidence of systemic or lymph node metastasis was identified at presentation, but local invasion of the peri-vulvar skin occurred within 1 month of treatment. Metastatic spread was suspected based on macroscopic appearance, location, and cytologic findings. At the time of last reevaluation, the perivulvar lesions were static in size and number and were not causing any clinical signs. Furthermore, despite the absence of clinical signs suggestive of systemic or regional metastasis, progression could not be assessed because repeat screening was declined by the owner.

The patient was alive 1 year after diagnosis and 11 months after commencement of treatment. Median survival time of SCC varies with anatomic location and has been reported for SCC arising in the skin, tonsils, oral cavity, nasal planum, and male external genitalia as 1004 days, 179-243 days, 365 days, 453 days, and 356-660 days, respectively.^1^^,^^11^^,^^13^^,^^14^^,^^25^ The prognosis for vulvovaginal SCC in dogs is poor, with one affected dog surviving for 8 months before euthanasia, and a second surviving at least 4 months before being lost to follow-up.^4^

Lapatinib was well tolerated in our patient with no clinical adverse effects being identified. However, an initial increase in ALT activity did require treatment cessation for 16 days and hepato-protectant antioxidant therapy using Denamarin and α lipoic acid for 190 and 57 days, respectively. Supportive treatment resulted in a rapid normalization of ALT activity within 2 weeks and ALT activity that remained normal after restarting lapatinib treatment. Serial serum biochemistry monitoring is recommended, and supportive hepato-protectant treatment can be considered when using lapatinib.

Lapatinib treatment was well-tolerated and effective in our case, resulting in a longer survival time than curative intent surgical excision. However, additional studies including prospective clinical trials are needed to determine the efficacy of lapatinib in the treatment of SCC as a sole therapeutic, adjuvant, or neo-adjuvant therapy.

## Data Availability

The data that supports the findings in this paper is available upon request to the corresponding author (M.J.V.).

## Off-label antimicrobial declaration

Authors declare no off-label use of antimicrobials.

## Institutional animal care and use committee or other approval declaration

Authors declare no institutional animal care and use committee or other approval was needed.

## Abbreviations

SCC: squamous cell carcinoma
DFI: disease free interval
EGFR: epidermal growth factor receptor
HER2: human epidermal growth factor receptor 2
IHC: Immunohistochemistry
